# Stable Dopamine-Signaling mRNA Co-Expression in the Substantia Nigra Is Deregulated in Pathological Conditions, but Not in Dopamine Transporter Knockout Rats †

**DOI:** 10.3390/biom15081117

**Published:** 2025-08-03

**Authors:** Anastasia N. Vaganova, Zoia S. Fesenko, Anna B. Volnova, Raul R. Gainetdinov

**Affiliations:** Institute of Translational Biomedicine, St. Petersburg State University, Universitetskaya nab. 7/9, 199034 St. Petersburg, Russia; a.n.vaganova@spbu.ru (A.N.V.); z.fesenko@spbu.ru (Z.S.F.); a.volnova@spbu.ru (A.B.V.)

**Keywords:** dopamine, dopamine plasma membrane transport proteins, substantia nigra, models, animal, gene expression profiling, transcriptome, high-throughput nucleotide sequencing, RNA-seq, RNA, messenger

## Abstract

Dopamine transporter (DAT) mutations are associated with neurological and psychiatric diseases, and DAT gene knockout in rats (DAT-KO) provides an opportunity to evaluate the DAT role in pathological conditions. We analyzed DAT expression and co-expression with other genes in the substantia nigra and striatum in public transcriptomic data represented in the GEO repository and then estimated the identified DAT co-expression pattern in DAT-KO rats by RT-PCR. In silico analysis confirmed DAT expression in the substantia nigra and absence of DAT mRNA in the striatum. Also, DAT is co-expressed with genes involved in dopamine signaling, but these associations are disrupted in dopamine neuron-damaging conditions. To estimate this co-expression pattern when DAT expression is lost, we evaluate it in the substantia nigra of DAT-KO rats. However, in DAT-KO rats the associations between genes involved in dopamine signaling were not disturbed compared to wild-type littermates, and tyrosine hydroxylase expression upregulation in the substantia nigra of these animals may be considered as compensation for the loss of dopamine reuptake. Further studies of expression regulation in dopamine neurons of DAT-KO rats may provide valuable information for compensatory mechanisms in substantia nigra dopaminergic neurons.

## 1. Introduction

Termination of synaptic dopamine (DA) action is primarily achieved by its rapid dopamine transporter (DAT)-mediated reuptake from the synaptic cleft into presynaptic terminals [1]. Its activity enables regulation of the dopaminergic signals’ duration and retention of DA in the presynaptic terminals [2]. DAT deregulation or deficiency is associated with psychiatric and neurodegenerative disorders. Study of postmortem substantia nigra (SN) samples revealed upregulation of DAT gene expression in the schizophrenic patients’ samples compared to control subjects [3]. In consistency with these results, higher DAT availability in schizophrenia patients in midbrain, striatal, and limbic regions was demonstrated [4]. DAT density also grows in the striatum of bipolar disorder patients compared to controls, both in the euthymic [5] and maniacal states [6]. In the meantime, DAT polymorphisms that trigger anomalous DA uptake [7,8] may be associated with neuropsychiatric disorders, including bipolar disorder, autistic spectrum disorders (ASD), or attention-deficit/hyperactivity disorder (ADHD) [8,9,10].

Striatal DAT binding decrease is identified in Parkinson’s disease (PD) and reflects axonal dysfunction of DA neuron projections of substantia nigra (SN) [11]. PD patients had reductions in SN volume [12,13], decreased intracellular DA content, and density of dopaminergic projections in the striatum [14]. Currently, DAT imaging is applied in the diagnosis and differential diagnosis of PD, atypical Parkinsonian syndromes, and other neurological disorders [15,16]. Imaging is also helpful to reveal the dopaminergic dysfunction in presymptomatic subjects at the prodromal stage of disease [16] and to monitor the disease progression [17,18]. However, these approaches visualize dopaminergic presynaptic ligand binding in the striatum, where DAT is located on presynaptic terminals, but not in the SN, where its mRNA is expressed in DA neuron bodies [16].

SN DA neurons possess long and highly branched unmyelinated axons [19]. One single human SN neuron could have 1 million to 2.5 million striatal synaptic sites [19]. DA neuron degeneration in PD is initiated in distal axons and proceeds retrograde [20], with loss of DAT-containing terminals in the early stages of disease. Thus, DA signaling deficiency in the SN is responsible for decreased locomotor activity in PD and Parkinsonism [21]. As a result, the correlation between DAT binding in the striatum and SN neuron loss may be low, especially in early stages of disease [22,23]. The data for molecular changes in the SN of PD patients also seems to be controversial. Previously, comparative analysis of microarray-generated datasets represented in the Gene Expression Omnibus (GEO) repository represented the low reproducibility of differential gene expression analysis in SN samples from PD patients and non-PD subjects [24]. This discrepancy covers DAT mRNA expression, which is downregulated only in four of eight datasets included in the analysis [25].

DAT knockout (DAT-KO) in mice and rats results in hyperactivity, repetitive non-goal-directed behavior, sensory gating deficits [26], impaired ability to perform learning tasks, and slower growth rate [1,27,28]. On the whole-brain level, DAT-KO rats exhibited decreased information processing and transmission [25]. Meanwhile, the decrease in body weight [1,28,29] and increased locomotor activity in DAT-KO rodents are variable and depend on the background [29,30,31]. Since the identified associations between DAT dysfunction and neuropsychiatric disorders, DAT-KO animals are considered the models of such diseases as ADHD [32,33,34], obsessive-compulsive disorder (OCD), OCD-ADHD comorbidity [35], and schizophrenia [4,33,36,37], and can strongly influence motivational behavior [38,39]. At the same time, DAT-KO rodents are considered as models of infantile Parkinsonism-dystonia, later renamed as Dopamine Transporter Deficiency Syndrome (DTDS) [38] and more prone to motor deficits, tremor, and akinesia after DA synthesis inhibition [40]. Importantly, a significant number of DAT-KO mice (30–40%) sporadically develop a DA-dependent progressive locomotor disorder characterized by dyskinetic movements, paralysis, and eventual death in several days [1].

Despite the growing body of data for the disturbance of brain functioning in DAT-knockout rats, the molecular background of the identified features needs further investigation. DAT-KO rats, with a knockout of the DAT gene, allow us to assess the role of DAT in the development of PD and in the molecular mechanisms of predisposition to Parkinsonian conditions. The aim of this study was to evaluate the functional association of the DAT gene in the substantia nigra as well as the stability of these associations in DA neuron-damaging diseases and DAT-KO rats. To this purpose, we identified the major DAT functional associations in SN using the public open-access transcriptomic data. Currently, this approach is considered valuable and useful for studies of the molecular ground of diseases [41,42]. Then, we estimate the expression of identified genes in the rats’ SN when DAT expression is lost or lowered by RT-PCR to evaluate the potential of these animals as models of human diseases.

## 2. Materials and Methods

### 2.1. Public Resources and Databases

The RNAseq data were mined from the public database Gene Expression Omnibus (GEO) [43] from NCBI. We used the terms “substantia nigra” and “midbrain” and the filter “Expression profiling by high-throughput sequencing” for the method. The inclusion criteria were, (1) at least 10 SN samples from the control study group; (2) the datasets represent the expression profiles of native samples or DA neuron fractions; data for single-cell and cell that do not express DAT were excluded; and (3) transcriptomic data are represented in raw counts. Additionally, we included in the analysis the dataset GSE136666, which represents data for 5 PD patients and 5 non-Parkinsonic subjects. We used these data for the comparative analysis but not for the study of correlations because of the low number and high heterogeneity of the studied groups. After the exclusion of non-relevant datasets, three RNA-seq datasets listed in Table 1 were included in the review.

Additionally, we searched the GEO database for the mouse striatal transcriptomic data received by RNA sequencing. We used the terms “striatum”, “globus pallidum”, “nucleus accumbens”, “nucleus caudatus”, and “putamen”. Filters “Expression profiling by high throughput sequencing” for the method and “Mus musculus” for the organism(s) were applied. Data were selected with the same inclusion criteria as described above. Only samples from wild-type, intact animals were selected for the analysis. The selected striatal datasets are listed in Table 2.

**Figure 1:**
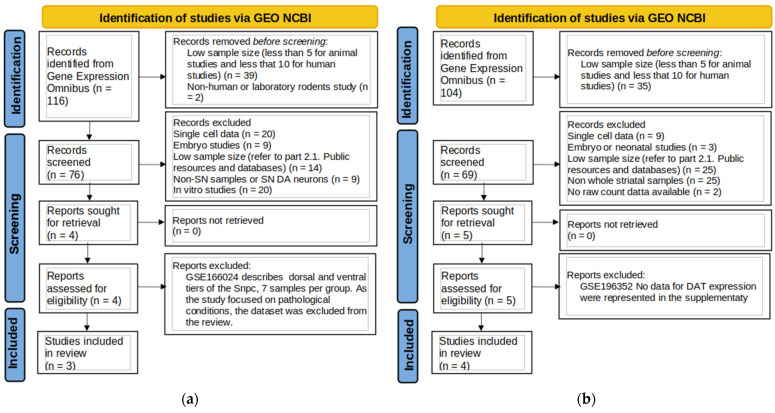
Flowchart of the data search for substantia nigra (**a**) and striatum (**b**).

The flowcharts (Figure 1a,b) represent the flow of dataset selection and the application of inclusion criteria.

### 2.2. Data Normalization and Statistical Analysis

Raw counts for selected datasets were downloaded from the GEO NCBI repository and CPM (count per million) normalized by the edgeR package [45]. CPM values above the threshold level of 0.5 were considered positive (following the cut-off values described elsewhere [45,46]). Expression data were visualized by the ggplot2 package [47]. 

Differentially expressed genes (DEG) were identified by the edgeR log likelihood ratio test [45] after the filtration by function filterByExpr with default arguments. Data were normalized by the calcNormFactors edgeR command using the trimmed mean of M-values method (TMM). *p*-values were adjusted for multiple testing corrections by the Benjamini–Hochberg method. Genes were considered as differentially expressed if adjusted *p*-values (*p*adj) < 0.05.

### 2.3. Measurement of Co-Expression and Functional Analysis

Data for different study groups were filtered independently. DAT co-expressed genes were selected by Spearman’s correlation coefficient (*p* < 0.05; the ρ cut-off values are described below in Results). The chi-square test was applied to evaluate the differences in the proportion of the number of genes whose expression was positively or negatively correlated with DAT mRNA levels between subgroups.

Gene Ontology (GO) [48] semantic similarity in identified gene clusters was calculated by Wang’s method in the GOSemSim (v.2.34.0) package employing the “Biological process” (BP) GO terms [49]. Pairwise semantic similarities were calculated with the mgeneSim function. The difference in semantic similarity scores in different gene clusters was estimated by the Kruskal–Wallis test and compared with the results of the same analysis performed on the random gene set. The results were visualized by ggplot2 (v.3.5.2.)

The clusters of DAT co-expressed genes, identified in different study groups, were compared to each other. BP GO term and Kyoto Encyclopedia of Genes and Genomes (KEGG) pathway enrichment analysis was performed in gene clusters identified as described above, applying the clusterProfiler (v. 4.16.0) Bioconductor R package [50], by the “enrichGO” and “enrichKEGG” functions, respectively. Considering the informativity of gene–disease associations identification [51,52,53], Disease Gene Network (DisGeNET) [54] ontology enrichment was performed with enrichDGN function in the DOSE (v.4.2.0) Bioconductor R package [55]. The function “compareCluster” in clusterProfiler (v. 4.16.0) was applied for a comparative enrichment study in several gene clusters. We considered significant enrichment results only for GO biological process terms, DO terms, or KEGG pathways with a false discovery rate value of <0.05. Visualization of the functional enrichment result was performed by the “dotplot” function in the enrichplot (v. 1.28.2) R package based on ggplot2 graphics.

Correlations were compared with the Zou confidence intervals method in the cocor (v. 1.1–4) R package [56].

### 2.4. Animals

Considering the minimal sample size to identify 50% expression changes for variably expressed genes [57,58], SN samples were collected from 15 adult male rats: DAT knockout (DAT-KO, n = 5), DAT-KO heterozygous (DAT-Het, n = 5), and wild-type (WT, n = 5) male rats aged 5 months. Rats were bred following a HET–HET breeding scheme. Each genotype group was formed based on genotyping results. Animal genotyping was performed according to the previously described protocol [26]. Rats were assigned to groups based on their genotype. All experimental procedures and protocols were approved by the Ethics Committee for Animal Research of Saint Petersburg State University, St. Petersburg, Russia, no. 131-03-2 of 28 of February 2025. Rats were maintained in IVC cages (RAIR IsoSystem World Cage 500; Lab Products, Inc., Seaford, DE, USA) with free access to food and water, 50–70% relative humidity, and a 12 h light/dark cycle (light from 9 a.m.) at a temperature of 22 ± 1.0 °C.

Rats were euthanized by decapitation with prior anesthesia with isoflurane gas.

### 2.5. RNA Isolation, Reverse Transcription, and Quantitative Polymerase Chain Reaction (qPCR)

Total RNA was isolated from tissue samples using ExtractRNA (Evrogen, Moscow, Russia) according to the manufacturer’s guidelines. RNA concentration was estimated by NanoDrop 2000 (Thermo Scientific, Waltham, MA, USA). For reverse transcription, 300 ng of each RNA sample was taken. Reverse transcription was performed using the MMLV RT kit (Evrogen, Moscow, Russia) in accordance with the manufacturer’s recommendations.

Differences in specific mRNA levels were assessed by qPCR. The primer sequences used in this study are listed in Table 3. Each cDNA sample was run at least twice using the CFX96 Touch Real-Time PCR Detection System (Bio-Rad, Hercules, CA, USA).

The expression of *Ddc*, *Drd2*, *Vmat2*, and *Th* was estimated by qPCR with qPCRmix-HS SYBR (Evrogen, Moscow, Russia). To assess specificity, amplification products were subjected to melting curve analysis.

Relative expression quantification was performed using the 2^−ΔΔCt^ method. During the analysis, the following parameters were calculated for each reaction: the Ct value; the ΔCt value for replicates, which was calculated by subtracting the housekeeping gene *Pkg2* Ct value from the Ct value for the gene of interest; the mean Ct value for each gene in the WT group, which was calculated and subtracted from ΔCt to receive ΔΔCt; and then the 2^−ΔΔCt^ value was calculated. Each test was performed in triplicate; the mean values were used for further analysis. After the Shapiro–Wilk normality test, the differences in the normalized expression levels were assessed using a *t*-test (for normally distributed values, i.e., *p* > 0.05 for all groups) or a Mann–Whitney U test. Thus, *Th* expression differences were estimated by pairwise *t*-test with Holm–Bonferroni adjustment; all other gene expression levels were compared with the U-test with Holm–Bonferroni adjustment.

## 3. Results

### 3.1. DAT Expression in the Mouse Substantia Nigra and Its Association with Other Components of Dopaminergic Signaling

**Figure 2:**
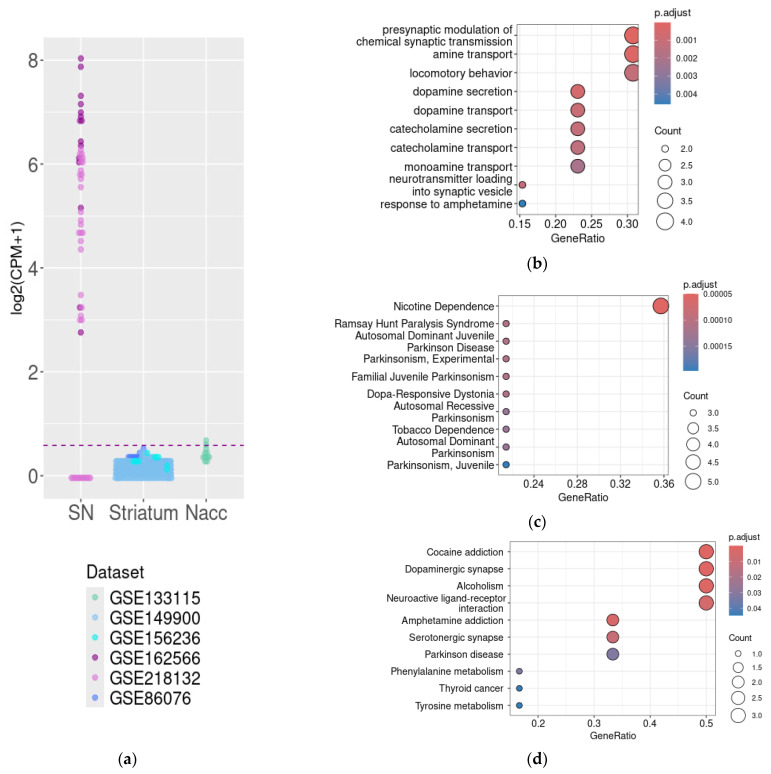
DAT expression in mouse substantia nigra, striatum, and nucleus accumbens. The CPM-normalized DAT mRNA expression in the datasets included in the analysis, with a cut-off level of 0.5 CPM represented by the dotted line (**a**). Functional analysis of genes co-expressed with DAT in the substantia nigra by GO Biologic Process term enrichment (**b**), DisGeNET term enrichment (**c**), and KEGG (**d**). CPM—count per million, Nacc—nucleus accumbens, and SN—substantia nigra.

Two mouse transcriptomic datasets generated by RNA sequencing were included in the analysis. Congruent patterns of DAT gene expression were identified in both datasets. In the GSE162566, the mean DAT gene expression level was 107.29 +/− 71.60 CPM (from 5.9 to 253.8 CPM), and in the GSE218132, DAT gene mRNA was expressed in 69% of samples at the mean level of 27.7 +/− 27.36 CPM (from 0 to 75.6 CPM) (Figure 2a). The identified discrepancy may be related to the samples’ properties, because the GSE162566 dataset represents the results of a whole tissue study, and the GSE218132 dataset consists of data for isolated cell nuclei sequencing. In contrast, no DAT gene expression above the cut-off level of 0.5 CPM was identified in the mouse striatal samples, except in two samples of nucleus accumbens (GSE133115, Figure 2a).

For the further analysis of DAT functional relations in the SN, we analyzed genes whose expression levels correlated with DAT mRNA expression levels in both the GSE162566 and GSE218232 datasets. Genes were selected by Spearman’s correlation coefficient (cut-off values were ρ > 0, *p* < 0.05 for both datasets).

The DAT mRNA co-expressed gene cluster selected as described above included only 14 genes, which are reproducibly co-expressed with the DAT gene in the mouse SN, including gene coding proteins involved in DA signaling, including dopamine receptor *Drd2*, vesicular monoamine transporter *Vmat2*, L-amino acid decarboxylase, and L-AADC (or dopamine decarboxylase, *Ddc*). The GO BP and KEGG pathway (Figure 2d) enrichment analysis identified co-expression of DAT with genes involved in biological processes related to DA signaling. Together with DAT, products of these genes are involved in four closely related steps, i.e., dopamine synthesis, feedback signaling loop, reuptake, and storage. In particular, GO BP enrichment identified genes involved in synaptic activity and neurotransmitter transport (Figure 2b), and KEGG pathway enrichment identified the association of DAT co-expressed genes with the dopaminergic synapse and neuroactive ligand-receptor interactions (Figure 2d). In line with these results, the associations with genes involved in the pathogenesis of diseases associated with the deregulation of DA signaling were identified by DisGeNET enrichment (nicotine addiction and Parkinsonism, Figure 2c) and KEGG-pathway enrichment (cocaine addiction, alcoholism, and Parkinson’s disease, Figure 2d).

### 3.2. DAT Expression in the Mouse Substantia Nigra Is Stable in Damaged Mouse Substantia Nigra, but Its Functional Interactions Undergo Significant Changes

**Figure 3:**
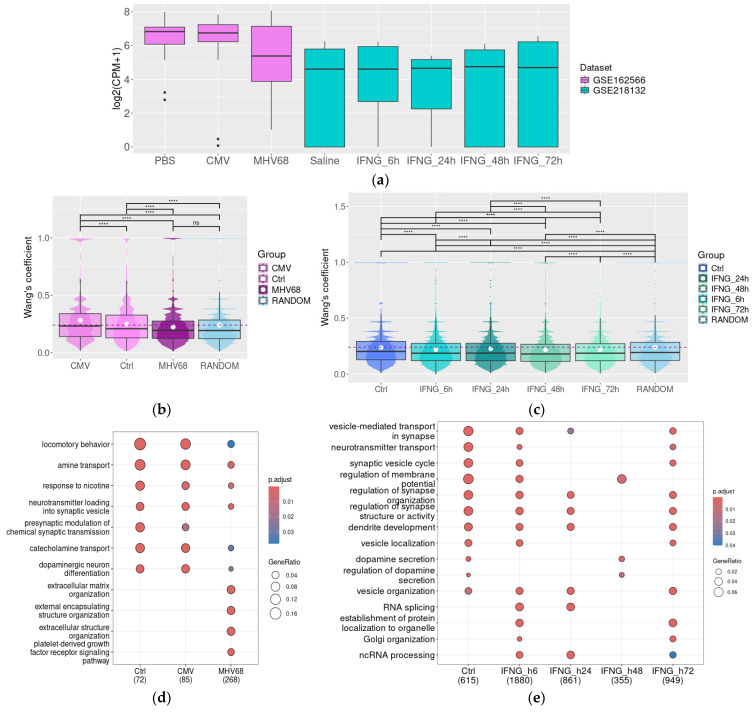
In mouse models, DAT expression is stable in pathologic conditions, but its co-expression landscape may be disturbed. DAT gene expression levels in the substantia nigra damaged by prenatal CMV or HNV68 infection in mice (GSE162566) or by IFN-γ injection (IFNG, neuron nuclear fraction, GSE218132); the upper, middle, and lower lines of boxplots represent first quartiles, medians, and third quartiles (**a**). Semantic similarity of GO BP for genes co-expressed with DAT in the intact substantia nigra and the substantia nigra after prenatal CMV or HNV68 infection (GSE162566) (**b**), or IFNG injection, 6 h, 24 h, 48 h, and 72 h post-injection (GSE218132); Wang’s coefficient characterizes the similarities between genes for each possible gene pair in the selected list based on their GO term characteristics (**c**), GO term enrichment for genes co-expressed with DAT in the intact substantia nigra and in the substantia nigra damaged by prenatal infections (GSE162566) (**d**), or by IFNG injection (GSE218132) (**e**). CMV—mouse cytomegalovirus, CPM—count per million, IFNG—interferon gamma, IFN-γ, and MHV68—murine gamma herpesvirus-68, ****—*p* < 0.0001.

Mouse transcriptomic datasets included in the analysis demonstrate the effect of several pathogenic factors on the dopaminergic system transcriptome. GSE162566 was generated for the study, which highlights dams’ Herpesviridae infections on the DA neurons in offspring and demonstrates that in adult offspring of murine gamma herpesvirus-68 (MHV68)-infected dams, there was significantly decreased expression of genes linked to DA neurons and a reduced proportion of DA neuron genes in the midbrain [44]. Despite these results, the DAT gene expression level was not significantly reduced (*p* > 0.05) in the offspring of MHV68- or murine cytomegalovirus (CMV)-infected dams (Figure 3a). However, because of the deleterious effect of maternal infection, the functional associations between DAT and other genes are significantly disturbed [44].

First, we select genes that are co-expressed with DAT in different groups, applying the cut-off level ρ > 0.75, *p* < 0.05. The semantic similarity of GO BP terms in the selected gene cluster demonstrated the dramatic loss of functional associations between genes co-expressed with DAT in SN in offspring of MHV68-infected dams (the mean Wang’s coefficient value does not differ from the mean Wang’s coefficient value in 1000 randomly selected genes, Figure 2b). In contrast, in the control group and offspring of CMV-infected dams, DAT-co-expressed genes demonstrated closer functional relations, with the mean value of Wang’s coefficient above that in the random gene cluster (n = 1000). In line with this result, GO BP enrichment demonstrated a broad convergence between functional characteristics of DAT-co-expressed genes in the control group and offspring of CMV-infected dams. The enriched GO BP terms included terms associated with dopaminergic neuron functions like locomotor behavior, catecholamine transport or neurotransmitter loading into synaptic vesicles, presynaptic modulation of chemical synaptic transmission, and dopaminergic neuron differentiation. In contrast, in the SN from MHV68-infected dams, the identified associations are less pronounced or lost (Figure 3c) along with the development of new functional associations, which were not identified in intact mice. These associations include co-expression of the DAT gene with genes involved in organic acid synthesis and extracellular matrix organization.

The dataset GSE218132 represented the transcriptome of isolated nuclei of dopaminergic neurons from the midbrain after the IFN-γ exposure (Figure 3a). The original study found no evidence of DA-neuronal degeneration or toxicity after such treatment and identified a stable expression of DA neuron-specific mRNAs like *Th*, *DAT*, and *Ddc* [59]. We also selected *DAT*-co-expressed genes applying the cut-off level ρ > 0.75, *p* < 0.05 for the control group and applying the cut-off level ρ > 0.95, *p* < 0.05 for IFN-γ-treated groups because gene clusters selected with less strict cut-offs were too large (i.e., 1500 or more genes per cluster). In contrast to the tendency identified in whole SN samples represented in GSE162566, we could not identify clusters with a mean Wang’s coefficient value for GO BP semantic similarity higher than the mean Wang’s coefficient value in the random gene set (n = 1000, Figure 2c). Possibly, this discrepancy in the semantic similarity analysis results mirrors the different sample nature in these datasets.

Despite the low semantic similarity in the identified gene clusters, GO BP enrichment analysis revealed significant over-representation of genes involved in synapse function, dopaminergic neurotransmission, and neurotransmitter transport in genes co-expressed with DAT both in saline-treated midbrain and in the midbrain samples following IFN-γ exposure at all time points (i.e., 6 h, 24 h, 48 h, and 72 h post-injection). The associations partially disappear at 24 and 48 h after injection but are restored at 72 h post-IFN-γ injection (Figure 3e).

### 3.3. The Association Between DAT and Other Genes Involved in Dopamine Synthesis and Signaling Is Destroyed in Parkinson’s Disease

**Figure 4:**
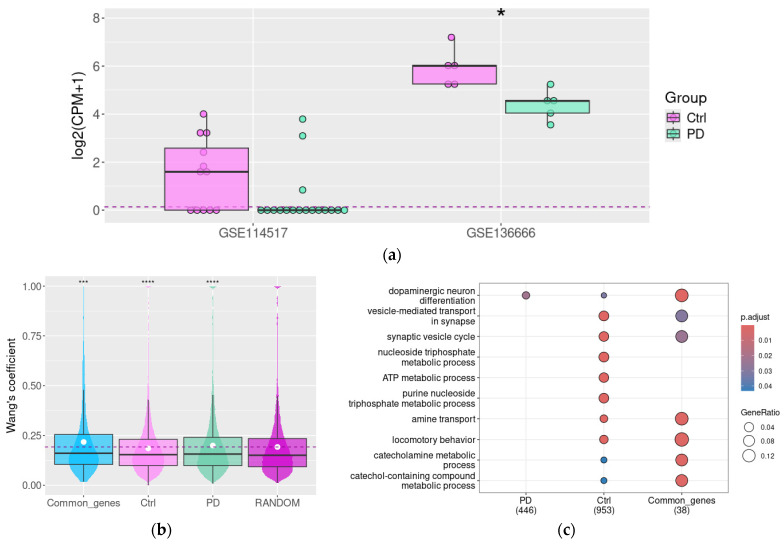
Functional associations between DAT and other genes in healthy aged subjects and patients with Parkinson’s disease. The DAT gene expression level in PD patients and control groups (in GSE114517 and GSE136666), *—*p* < 0.05; the upper, middle, and lower lines of boxplots represent first quartiles, medians, and third quartiles (**a**), Semantic similarity in DAT co-expressed genes cluster in PD patients’ substantia nigra samples compared to the non-demential control group and random gene set (in GSE114517); Wang’s coefficient characterizes the similarities between genes for each possible gene pair in the selected list based on their GO term characteristics (**b**). GO BP term enrichment for genes co-expressed with DAT in the substantia nigra of PD patients and control non-demential subjects (in GSE114517) (**c**); Ctrl—control (non-demential) group, and PD—Parkinson’s disease patients. ***—*p* < 0.001, ****—*p* < 0.0001 compared to random gene set.

Two human RNA-generated datasets (Table 1) available in the GEO repository were included in the present study. Both datasets represent the comparative study of SN from patients with PD and matched controls. As the authors of the dataset GSE114517 described in their paper, the dopaminergic neuron marker TH and different components of synaptic pathways, but not DAT expression, are deregulated in PD SN samples [60]. In contrast, in a study based on the GSE136666 dataset, authors identified the significant downregulation of DA in PD samples [24]. Under these data, we identified pronounced DAT downregulation in PD samples in this dataset (*p*adj < 0.05, Figure 4a).

The DEG analysis did not demonstrate a statistically significant decrease of DAT gene mRNA expression in PD patients in GSE114517. However, seven (58%) of non-PD samples and only three PD SN (18%) samples were DAT-positive in this study (Figure 4a). Despite the low number of DAT-positive samples in the PD group, we tried to identify DAT co-expressed genes by the same approach as described above, i.e., applying the cut-off level ρ > 0.75, *p* < 0.05. However, the number of genes co-expressed with DAT in PD patients’ samples was too low for further analysis (only six genes), so we selected all DAT co-expressed genes for the analysis, applying the milder cut-off level ρ > 0.5, *p* < 0.05.

We estimated the GO terms’ semantic similarity in DAT-co-expressed gene clusters in the SN of PD patients and control subjects. In control subjects, the mean Wang’s similarity coefficient in this gene cluster was lower than in the random gene set. Instead, in PD subjects, the mean Wang’s coefficient in DAT co-expressed genes exceeds the mean value in random genes (n = 1000, Figure 3b). Furthermore, the gene group was still co-expressed with DAT both in control and Parkinson’s subjects. The functional similarity in these gene clusters also was higher than in the random gene set.

Like the tendency identified in mouse samples, the DAT-co-expressed gene cluster in control subjects is enriched with genes involved in synaptic vesicle transport (Figure 4c). Also, we identified the association between DAT expression and the expression of genes encoding proteins that are involved in purine metabolism and aerobic respiration. In samples harvested from the PD group, all these associations are partially destroyed (as demonstrated by the analysis of common genes co-expressed with DAT in both conditions), except the enrichment of the DAT co-expressed gene cluster by the term dopaminergic neuron differentiation.

DAT co-expression with DRD2, VMAT2, and DDC identified in mouse samples is also retained in human SN. It is noteworthy that in both PD patients and non-demented controls, DAT is co-expressed with transcriptional factors FOXA1/2, LMX1A/B, and EN2 involved in dopaminergic neuron development. Meanwhile, in the control group, it also co-expressed with transcriptional factors HPRT1, EN1, and Wnt, signaling pathway regulator RSPO2. These associations were not identified in the PD group; at the same time, in these subjects, DAT was co-expressed with other transcriptional factors involved in dopaminergic neuron development, including PITX3, OTX2, and NR4A2.

### 3.4. Co-Expression of Key Components of DAT-Co-Expressed Genes in Damaged SN Samples

**Figure 5:**
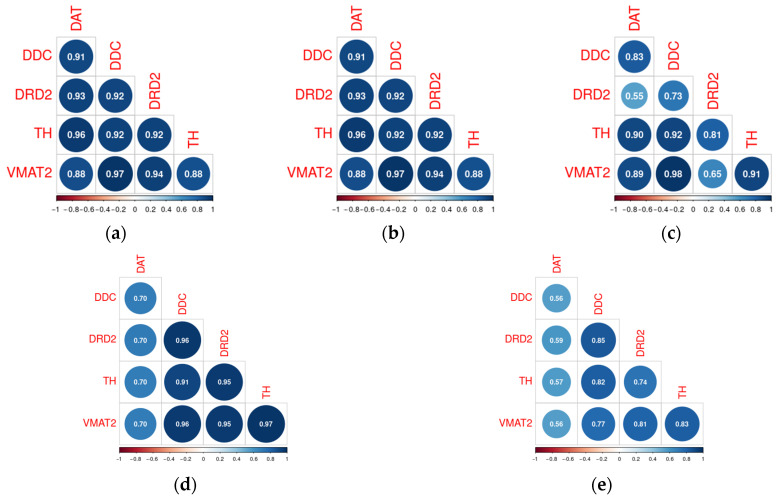
Correlations between DAT, DDC, DRD2, VMAT2, and TH in the normal mouse substantia nigra (**a**), substantia nigra damaged by prenatal CMV (**b**) or MHV68 (**c**) infection in mice (GSE162566), normal human substantia nigra (**d**), and substantia nigra of PD patients (**e**) (GSE114517).

As we previously identified, in the intact adult SN, the DAT gene is co-expressed with other components of the dopaminergic signaling system, including DRD2 and VMAT2 genes, both in mice (GSE162566, Figure 4a) and humans (GSE114517, Figure 5d). We also included the DA neuron marker TH in this part of the study to estimate disturbances of the co-expression of all components of dopamine synthesis and storage in available data.

The changes in the co-expression profile among genes involved in DA signaling varied across the different datasets included in the analysis. The associations between genes involved in DA signaling remain stable in the SN CMV-infected dams’ offspring (Figure 5a,b), but become disrupted in offspring of MHV68-infected dams (Figure 5c) and patients with Parkinson’s disease (Figure 5d,e). In offspring of MHV68-infected dams, the loss of correlations between *Drd2* and *Vmat2*, *Ddc*, DAT, and *Th* reaches a significant level (*p* < 0.05). At the same time, in patients with PD, only the loss of correlation between DAT and the other studied genes reaches statistical significance (*p* < 0.05).

### 3.5. Stable Co-Expression of Key Components of DAT-Co-Expressed Genes in DAT-KO Rats

**Figure 6:**
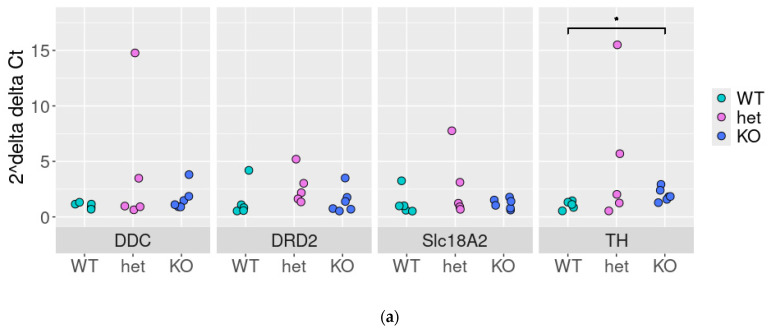

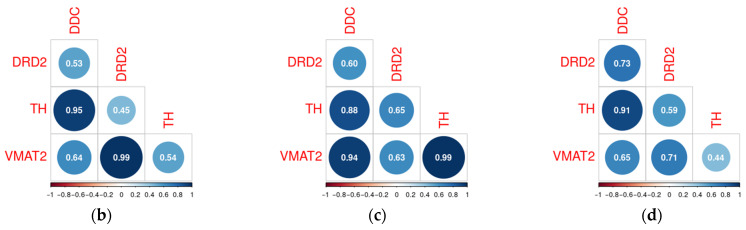
Expression and correlations between *Ddc*, *Drd2*, *Vmat2*, and *Th* in rat lines lacking the dopamine transporter substantia nigra: qPCR results in WT, DAT-Het, and DAT-KO rats: each dot represents expression level in a sample, and expression levels of genes were normalized to the expression of the housekeeping gene *Pkg1* by 2^−ΔΔCt^ method as described above, *—*p* < 0.05 (**a**), correlations—between genes expression (in Ct) in WT (**b**), DAT-KO heterozygous (**c**), and DAT-KO rats (**d**).

Thus, we tested the stability of these associations (except DAT, which is known to be downregulated in DAT-Het and completely lost in DAT-KO rats) in DAT-WT, DAT-KO, and DAT-Het rat SN samples by qPCR. We identified the slight (~2-fold change, Figure 6a) up-regulation of TH expression in DAT-KO rats (*p* < 0.05, Figure 6a). Meanwhile, the upregulation of *Th* mRNA expression levels in DAT-Het rats compared to DAT-WT did not reach statistical significance (*p* > 0.05).

Despite some deregulation of correlations between studied genes in DAT-Het and DAT-KO rats, no significant differences in correlation profiles among these genes were revealed between the studied animal groups (Figure 6b–d).

## 4. Discussion

The dopamine transporter DAT is considered the selective marker of DA neurons [61,62]. Its deregulation, both in cerebral structures [22,26] and on the systemic level, as identified by the study of peripheral blood mononuclear cells, is associated with PD. The DAT protein was identified in dopaminergic axons, mainly in the mesencephalic dopamine neurons of the SN and the ventral tegmental area, striatum (including putamen and caudate nucleus), and nucleus accumbens, with much lower levels in the amygdala, hypothalamus, hippocampus, some thalamic nuclei, and neocortex [63]. Visualization of DAT binding with labeled ligands is considered a powerful tool for early diagnosis of PD and the monitoring of its progression [16,17,18]. Meanwhile, DAT mRNA was not detected in the midbrain DA neuron axons. At the same time, it is identified in DA neuronal soma and dendrites as another component of DA synthesis, release, and reuptake machinery [60,64]. The protein visualization also reveals that DAT is translated in the neuronal soma and transported between the midbrain and striatum by lateral diffusion in the plasmatic membrane [65]. In accordance with results previously described in the literature, we did not reveal DAT gene mRNA expression in the striatal samples in public transcriptomic data and concentrated our further analysis on the SN samples, where DAT mRNA was expressed.

As limited and heterogeneous data were available, we first identified genes co-expressed with the DAT gene in mouse samples, both in whole SN samples and fractionated DA midbrain neurons. We identified only a narrow, stable DAT co-expressed gene cluster, enriched with genes associated with dopamine signaling. The enrichment core includes the DRD2, DDC, and VMAT2 genes. The identified low number of positive correlations between DAT and other mRNA expressions may be associated with stable DAT mRNA levels, which are not prone to either physiological fluctuations or damaging impacts, like infections [44], inflammation [59], neurodegeneration [66], or aging [67]. So, DAT expression and activity seem to be regulated mainly at the post-transcriptional level [68] by alternative splicing [69], post-translational modifications, kinases, binding partners, and membrane rafts [70,71] against the background of stable mRNA transcription.

Despite the stable level of DAT expression in the SN samples from mice with prenatal CNS damage by maternal MHV68 infection, for mice suffering the injection of IFN-γ into the ventral midbrain, or patients with Parkinson’s disease, the quantity and composition of DAT co-expressed genes are considerably changed [59]. In GSE162566, as identified in the original study, dopamine neuron-related gene expression is suppressed in the SN in offspring of MHV68-infected dams [44]. DAT expression levels in SN are retained stably despite the reduction in the number of DA neurons. In the meantime, its co-expression pattern is disintegrated, and its mRNA co-occurrence with the mRNA of genes involved in synaptic function and neurotransmission becomes weaker. Instead, the new associations include co-expression with genes involved in organic acid biosynthesis and extracellular matrix. Notably, in PD brain tissue, including SN, extracellular matrix assembly and organization are significantly dysregulated [72,73], as was also confirmed in PD patients’ iPSC-derived dopaminergic neurons [74]. Thus, interaction between metabolic dysfunction, neuroinflammation, and extracellular matrix is a remodeling currently considered to be involved in PD progression [75]. However, based on the data available in GSE162566, it is difficult to interpret the identified co-expression as an association of DAT expression with a healthier SN state or, in contrast, with more pronounced neuron damage.

The dataset GSE218132 represents the effect of IFNG on dopamine neurons [59]. IFNG release from microglia regulates the expression/release of other cytokines and chemokines [76], recruits leukocytes, and disrupts the blood–brain barrier [77,78]. Neurons show no signs of cellular damage following IFNγ exposure in vitro [78]. The authors demonstrated that there was no evidence of IFNG-associated neurotoxicity or DA neuron degeneration in vivo. In all samples, neuron-specific mRNAs like Th, DAT, and Ddc were still expressed post-injection, and the identified effects seem to be reversible [59]. Following this result, the identified deregulation of the DAT-co-expressed genes profile in acute inflammatory conditions is reversable and also tends to recover in 72 h post-injection together with the general dopamine neuron expression pattern recovery.

Human study groups are more heterogeneous than animal models. The data of previous studies in different populations demonstrate the general trend-reduced DAT expression in SN samples of PD patients compared to matched non-PD groups [24,76]. However, these results are not reproduced in all microarray-generated public datasets [76], which were analyzed in published studies, or in two RNAseq-generated datasets [24,60], which we compare in the present study. Also, a previous meta-analysis of microarray-generated datasets from the GEO repository identified deregulation in the co-expression of genes involved in dopamine signaling associated with PD, including DAT, TH, VMAT2, and DRD2. We also revealed the disturbance of this orchestrated co-expression pattern in the PD samples’ transcriptome. Additionally, we identified deregulation of the same gene cluster in mouse samples in a model of prenatal CNS damage by maternal infection, which is considered a potential trigger to the chronic state of enhanced susceptibility to developing PD [44]. On the other hand, in PD, DAT-associated genes may shift towards compensatory expression focused on dopaminergic neuron differentiation rather than reflecting true network breakdown.

These three identified genes whose co-expression with DAT was deregulated in pathologic conditions in studied datasets were selected for further study in DAT-KO rats’ SN. In addition, we also included in the study another DA neuron marker, TH, considering previously identified TH and DAT co-expression in VTA [79] and its role in dopamine synthesis. The absence of DAT leads to an increased amount of basal extracellular DA levels in the striatum, SNc, and VTA in laboratory rodents, i.e., mice and rats [26,27,80], and a decrease of total striatal DA content [26]. The amplitude of DA release per pulse is lower in the basal ganglia of mice with DAT knockout [81] or disrupted DAT interaction with scaffold proteins [82]. Simultaneously, the downstream dopamine signaling is also deregulated after the DAT loss [1]. It increased mRNA levels encoding the D3 receptor and decreased mRNA levels for both D1 and D2 receptors in the caudate nucleus, putamen, and nucleus accumbens, despite the number of neurons not changing [1,27,28,83,84,85]. The loss of D2 receptors interferes with DA neurons’ regulatory mechanism in DAT knockout animals [27].

Previously, the study of DAT-Het mice revealed that DA tissue content and DA turnover were unchanged relative to wild-type mice, despite a 40% reduction in DAT protein expression. At the same time, in DAT-KO mice, DA turnover increased in all DA compartments [80]. The DA synthesis rate-limiting enzyme TH loss is identified in the DAT-KO mice striatum [1,26,80], whereas, in somatodendritic compartments of the SN and VTA, TH expression was unaffected [80] or slightly up-regulated, as identified in the present study. However, loss of TH protein in DAT-KO mice striatum is accompanied with higher TH phosphorylation at ser40, which increases its enzymatic activity [80]. In DAT-Het mice, h protein concentration increased in the striatum, but was unaffected in the SN [80]. Interestingly, we did not reveal significant deregulation of the DAT-co-expressed gene cluster in DAT-Het or DAT-KO rats. Thus, in DAT-knockout rats without symptoms of PD or Parkinsonism, the regulation of DA synthesis machinery on the transcriptional level is not deregulated. On the other hand, Th gene mRNA expression is upregulated in DAT-KO rats. The identified discrepancy may be the result of altered Th enzyme turnover in DAT-KO rodents or differences between rats and mice.

The mechanisms underlying previously described changes in DA neuron functioning in these animals need further study and seem to occur not at the transcription regulation level.

Our study has several limitations. Only a few datasets in the GEO repository were relevant for the study, so we were required to analyze the association between DAT and other genes in heterogeneous datasets. In the present paper, we compare and summarize transcriptomic datasets obtained in various laboratories when researching vastly different samples, i.e., postmortem human tissues, mouse whole-tissue SN samples, and neuronal nuclei isolated from mouse midbrain. This approach allowed us to identify the most stable associations, but we may lack some significant data, which is specific to each of these kinds of samples. So, because we concentrated on these conservative and pronounced associations, additional high-throughput investigations are still necessary to more precisely identify new associations between DAT and other gene expressions in the SN. Additionally, we used the qPCR method to estimate the co-expression of genes involved in DA signaling in the SN of rats. This method is considered sufficiently accurate to identify two-fold changes but may be less sensitive to small fluctuations. At the same time, the overall level of gene expression is determined by several factors, such as RNA stability, modifications, and translation, and needs to be estimated on different levels by distinct approaches.

The study included transcriptomic datasets obtained on the narrow number of heterogeneous samples in various laboratories, and completely standardizing them is not possible. Direct comparisons and unified conclusions based on such heterogeneous data are problematic and risk reducing the reliability of the interpretations. Thus, we focused on stable associations that were reproducible in different studies and circumstances, and that became disrupted in pathologically changed SN samples. This change covers, first of all, principal genes, which are necessary for DA synthesis and storage in DA neurons. At the same time, we did not find the deregulation of identified co-expression clusters in vivo in DAT-knockout rats, which are characterized by DA system dysfunction but do not demonstrate a Parkinsonism-like phenotype [27]. However, only male rats were included in this part of the study, which is a significant limitation for result generalizability.

The hyperactivity and stereotypic behavior are well described in DAT-KO rats [26]. Meanwhile, the molecular background of these features has not been yet discovered, so we did not suggest any specific mechanisms for compensation in DAT-KO rats to avoid speculations. The variability observed in our results across different models underscores the complexity of DAT’s role in these relationships. So, DAT-KO rats seem to be the model animals to study the molecular bases of DA-signaling stability in DA-neuron-damaging conditions, but not for its disruption following DA neuron injury. The identified associations and their functional significance need further studies and experimental validation. Also, as deregulation of identified gene clusters is involved in the pathogenesis of neurodegenerative changes in SN, it is necessary to understand if this disharmony may be corrected or compensated to improve patients’ condition.

## 5. Conclusions

Our analysis of publicly available data demonstrates stable DAT expression in SN samples, which seems not to be significantly correlated with any process in this structure except the dopaminergic signaling. Similar to previously published data, we observed that pathologic conditions damaging SN lead to deregulation of DA-signaling pathway components’ expression more than loss of their expression per se. These data raise the following questions: what regulatory pathways are destroyed in DA neuron degeneration, and could PD-related dysfunction of DA metabolism be compensated by these pathways’ modulation? Notably, we demonstrate that, despite the fact that the inhuman dopamine transporter deficiency is fatal, the loss of functional DAT expression in rats seems to be partially compensated by the up-regulation of the mRNA expression of rate—the limiting DA synthesis enzyme TH. The DAT-KO rats are characterized by pronounced changes of phenotype (low size, weight, high hyperactivity, impaired social and learning abilities, etc.), and loss of functional connectivity of SN with other brain structures, but do not demonstrate PD-like conditions, at least before the onset of old age, when SN were sampled. We have revealed that DAT-knockout animals exhibit distinct molecular characteristics compared to those observed in Parkinson’s disease patients and other animal models of substantia nigra damage. Additionally, some unidentified compensatory mechanisms provide stability of the orchestrated expression of other genes involved in DA synthesis and storage in DAT-KO rats. This raises the question of DAT-KO rats’ translational potential. Further study of expression regulation in SN is necessary to identify the way by which the loss of principal DA neuron marker DAT is compensated in DA neurons in DAT-knockout rats. High-throughput transcriptional and proteome analysis, and estimation of specific SN transcriptional factors like En1/2 and Foxa1/2 functional changes in DAT-KO rats, may be helpful to elucidate such compensatory mechanisms. The identified factors that contribute to DA-signaling stability may be considered as potential therapeutic targets. Such results may be translated into clinical practice for the treatment of diseases related to neurodegeneration in the SN.

## Figures and Tables

**Table 1:** RNAseq datasets representing substantia nigra transcriptomes included in the review.

Dataset ID	Title	n	Sequencing Platform	Samples Characteristics
GSE218132	Conserved and cell type-specific transcriptional responses to IFN-γ in the ventral midbrain	48	Illumina NextSeq 500, Illumina NovaSeq 6000	Isolated nuclei samples. Midbrain samples from C57BL/6J mice harvested in 6, 24, 48 or 72 h after intracranial IFNG injection.
		36		Isolated nuclei samples. Midbrain samples of vehicle control C57BL/6J mice.
GSE162566	Infection with herpesviridae in pregnant dams alters midbrain dopaminergic signatures in adult offspring	18	Illumina NovaSeq 6000	Murine tissue samples. 8 weeks of age offspring of dames infected by murine cytomegalovirus (for details refer [44])
		15		Murine tissue samples. 8 weeks of age offspring of dames infected by Murid herpesvirus 68 (for details refer [44])
		15		Murine tissue samples. Midbrain samples of intact animals.
GSE114517	Next generation sequencing reveals upregulation of the lncRNA LINC-PINT in the substantia nigra of human Parkinson’s disease patients compared to control donors	17	Illumina NextSeq 500	Human tissue samples. Substantia nigra from patients without Parkinson’s disease.
		12		Human tissue samples. Substantia nigra from patients without Parkinson’s disease (control group).
GSE136666	Transcriptomic profiling of substantia nigra and putamen in Parkinson’s disease	5	Illumina HiSeq 2000	Human tissue samples. Substantia nigra from patients without Parkinson’s disease.
		5		Human tissue samples. Substantia nigra from patients without Parkinson’s disease (control group).

**Table 2:** RNAseq datasets representing striatal transcriptomes included in the review.

Dataset ID	Title	n *	Sequence Platform	Samples Characteristics
GSE86076	High motivation for exercise is associated with altered chromatin regulators of monoamine receptor gene expression in the striatum of selectively bred mice	31	Illumina HiSeq 2000	15 striatal samples from randomly bred control lines and 16 samples from the lines selected for high voluntary wheel running (both male and female).
GSE133115	Chronic, Chemogenetic Stimulation of The Nucleus Accumbens Produces Lasting Effects on Binge Drinking and Ameliorates Alcohol-Related Transcriptional and Morphological Changes	11	Illumina HiSeq 2500	Nucleus accumbens from HDID-1 mice (both male and female)
GSE149900	Transcriptional profiling of striatum from 6-month-old mice heterozygous knockout for 52 genes and corresponding wildtype control mice	41	Illumina HiSeq 4000	Striatal samples from 6-month-old C57BL/6 mice (both male and female).
GSE156236	Transcriptional profiling of striatum and cortex from a LacO-Q140 inducible mouse model of Huntington’s disease with early and late mutant HTT lowering	10	Illumina NovaSeq 6000	Striatal samples from 6-month-old C57BL/6 mice (both male and female).

* the number of control, wild-type, non-treated samples included in the analysis.

**Table 3:** Primers and probes were used for quantitative RT-PCR in DAT-KO rat study.

Gene Symbol	Gene Name	F Primer	R Primer
*Ddc*	dopa decarboxylase	TGGCGTGGAGTTTGCAGATTCC	GTCCTGGTGACTGTGCCTCAGA
*Drd2*	dopamine receptor D2	CTTGAAGAGCCGTGCCACCC	TGTCTGCCTTCCCTTCTGACCC
*Vmat2*	solute carrier family 18 member A2	TGGGAAGGTGGCTATGTGCTCT	AGGAGTCCACCATCCCAATTGCA
*Th*	tyrosine hydroxylase	CGCTTCTTGAAGGAGCGGACTG	GCATGGCGGATATACTGGGTGC
*Pkg1*	protein kinase cGMP-dependent 1	CTGCACACAGAGCCCACAGTTC	AAGCCATTCCCCCACCGATGAT

## Data Availability

The data are available in the GEO database (https://www.ncbi.nlm.nih.gov/geo/ (accessed on 5 May 2025), the detailed information is listed in Table 1 and Table 2).
